# Simultaneous detection of dynamic calcium signaling and ERK activity in living cells

**DOI:** 10.52601/bpr.2023.230038

**Published:** 2024-10-31

**Authors:** Liting Zhang, Yan Mo, Shimin Mo, Ming Xia, Chaoliang Wei

**Affiliations:** 1 School of Basic Medical Sciences, Shenzhen University Medical School, Shenzhen University, Shenzhen 518055, Guangdong , China; 2 PKU- Nanjing Institute of Translational Medicine, Nanjing Raygen Health, Nanjing 210031, China

**Keywords:** Calcium signaling, ERK signaling, Living cells, Genetically-encoded probes

## Abstract

Calcium (Ca^2+^) is a universal second messenger in eukaryotic cells, and extracellular regulated protein kinases (ERK) are the core component of the mitogen-activated protein kinase (MAPK) signaling cascade. Both are involved in numerous physiological and pathological processes, such as organogenesis, tumorigenesis, proliferation, migration and apoptosis. Over the past decade, it has been found that calcium signaling can regulate the ERK activity through multiple mechanisms, and conversely, ERK signaling transduction can also affect the triggering and intensity of calcium signaling. However, there are few reports on how to perform real-time synchronous detection of these two signals. Here we described a method for dynamically and synchronously recording calcium signals and ERK activity in living cells, utilizing stable expression of multiple genetically-encoded probes and multi-channel synchronous detection technology using confocal microscopy. The protocol can be useful to address the spatiotemporal encoding dynamic mechanism of calcium signaling and ERK activity in single or multiple cells, and to reveal the interaction and causal characteristics of these two signals.

## INTRODUCTION

Calcium ion is an important second messenger and plays a critical role in regulating various functions of eukaryotic cells, such as proliferation, differentiation, autophagy, apoptosis and migration (Hammad and Machaca 2021; Smaili *et al*. 2013; Sukumaran *et al.* 2021). When cells are stimulated, the concentration of intracellular calcium ions increases, leading to the generation of corresponding calcium signals and activation of a series of downstream cascade reactions. The generation and regulation of calcium signals are mainly achieved by the release, transmission, and recovery of calcium ions. Calcium pumps and channels play a regulatory role in the transmission of calcium signals and maintain a steady state of cellular calcium concentration (Heredia *et al.* 2020).

ERK belongs to the MAPK family and are serine and threonine protein kinases. ERK is usually located in the cytoplasm and, after being activated, enters the nucleus to regulate gene transcription and expression (Guo *et al.* 2020). The basic signaling steps follow the MAPK tertiary enzyme cascade, consisting of upstream activator sequences MAP3K, MAP2K, and MAPK. In the ERK pathway, Ras serves as an upstream activating protein, Raf as MAP3K, MAPK/ERK kinase (MEK) as MAP2K, and ERK is MAPK, forming the Ras-Raf MEK-ERK pathway (Yang and Liu 2017). It was found that Ras-ERK signaling can regulate cell migration and invasion, and affect the occurrence and development of tumors (Samson *et al.* 2022).

It has been found that calcium signaling and ERK signaling are not two independent pathways, and in some cases, there are interactions between these two. Mottet et al. found that calcium acted upstream of ERK through the hypoxic signal transduction pathway, thereby enhancing the transcriptional activity of hypoxia inducible factor 1 (Mottet *et al.* 2002). The study by Grewal et al. showed that calcium influx induced by depolarization stimulated ERK activity in mouse adrenal pheochromocytoma cells (PC12 cells) and hippocampal neurons in a protein kinase A dependent manner (Grewal *et al.* 2000). Gudipaty et al. found that stretching epithelial cells generated calcium currents via the opening of Piezo1, activating ERK1 to trigger cyclin B transcription and initiate mitosis (Gudipaty *et al.* 2017). These results indicate that calcium signaling can regulate the ERK signaling pathway through different mechanisms. On the other hand, ERK activity can also affect calcium signals. For example, Rosado and Sage found that in the case of complete ERK inactivation, the number of calcium ions entering platelets decreased by 50% (Rosado and Sage, 2002); Klepeis *et al*. found that during the process of epithelial cell damage repair, epidermal growth factor (EGF) bound to receptors and then activates the ERK signaling pathway, thereby enhancing the intensity and duration of calcium waves (Klepeis *et al.* 2001); Studies have also found that transient receptor potential cation channel 6 (TRPC6) can regulate ERK signaling, thereby affecting calcium waves and ultimately accelerating wound healing in mice (Takada *et al.* 2014).

The widely used Ca^2+^ probes include small molecule indicators such as Fura-2, Indo-1, Fluo-3, and genetically-encoded Ca^2+^ indicators (GECIs) such as D1ER, GCaMP, CEPIA1er (Grienberger and Konnerth 2012). Due to the free nature of small molecule indicators, they are easily expelled and quenched by cells. While some indicators are easily embedded in organelles and lose detection ability, their cellular localization is also difficult to control. These issues to some extent limit the application of small molecule indicators for detecting long-term biological processes, although they can be used for rapid application (Palmer and Tsien 2006; Paredes *et al.* 2008; Roe *et al.* 1990). There are two types of GECIs, namely indicators involving Förster resonance energy transfer (FRET) and single fluorophore indicators. The main representative of the single fluorescent group GECI is the GCaMP family, which is composed of enhanced green fluorescent protein (EGFP), calmodulin, and calmodulin binding peptide M13. Since its discovery, GCaAMP has undergone multiple changes and the most commonly used GCaMP6 family includes three sensors with different kinetics: GCaMP6f, GCaMP6m, and GCaMP6s (Chen *et al.* 2013). Among them, GCaMP6f is the fastest protein-based calcium indicator for cytoplasmic free calcium, which can effectively detect the single action potential of mouse visual cortex pyramidal neurons and faithfully track dynamic sensory stimuli in single dendritic spines. Compared to GCaMP3 and GCaMP5G, GCaMP6f has the highest sensitivity and calcium affinity, which demonstrates that GCaMP6f is a very suitable sensor to detect calcium signaling in diverse cells.

Common methods for detecting ERK activity include antibody labeling, FRET or kinase translocation reporter (KTR) based probes. Due to cytotoxicity and high background issues, antibody labeling is not suitable for real-time dynamic detection of ERK activity in living cells. The advantage of FRET-based reporter proteins is that they can be used to measure kinase activity specific to subcellular regions (Miura *et al.* 2014). However, its efficiency strongly depends on the distance and direction between the donor and recipient proteins, which results in a very limited dynamic range of its response (Hirata and Kiyokawa 2016). KTR probe is a biosensor based on a single fluorophore, which can measure ERK activity through brightness changes caused by subcellular translocation (Nakamura *et al.* 2021). It is mainly composed of nuclear localization signal (NLS), nuclear output signal (NES), and fluorescent protein. The negative charge brought by substrate phosphorylation regulates its shuttle between the nucleus and cytoplasm, thereby regulating the subcellular distribution of reporter genes. Furthermore, when combined with H2B fusion fluorescent protein for nuclear segmentation, KTR probes can perform multiple imaging of kinase activity at the single-cell level (Maryu *et al.* 2016; Regot *et al.* 2014). However, KTR also has limitations in practical applications as it is unable to visualize subcellular kinase activity, making it unsuitable for studying kinases with local regulatory functions. Compared with FRET, the main determining factors for the dynamic range of KTR are the position of phosphorylation residues and the localization signal sequence, which endow the KTR probe with better activity and the ability to capture the inactivation dynamics. The advantages of the multiplexing and the relatively simple design process have made KTR widely popular among researchers (Kosugi *et al.* 2008; Kudo *et al.* 2018).

Given the importance of calcium signaling and ERK signaling pathways for cellular life activities, sensitive and accurate detection of them can help further explore the mechanisms and characteristics of cell behaviors. Based on the interaction between calcium signals and ERK activity, detecting them simultaneously can help us better study various cellular biological processes. Meanwhile, the genetically encoded fluorescent proteins suitable for live cell experiments lay a good foundation for the synchronous detection of both signals. Therefore, this protocol described how to simultaneously use GCaMP6 and ERKKTR to detect calcium signaling and ERK activity in living cells, which can be widely applied to explore characteristics and connections of the two signals in a single or population of various eukaryotic cells.

## OVERVIEW OF THIS PROTOCOL

**Figure 1:**
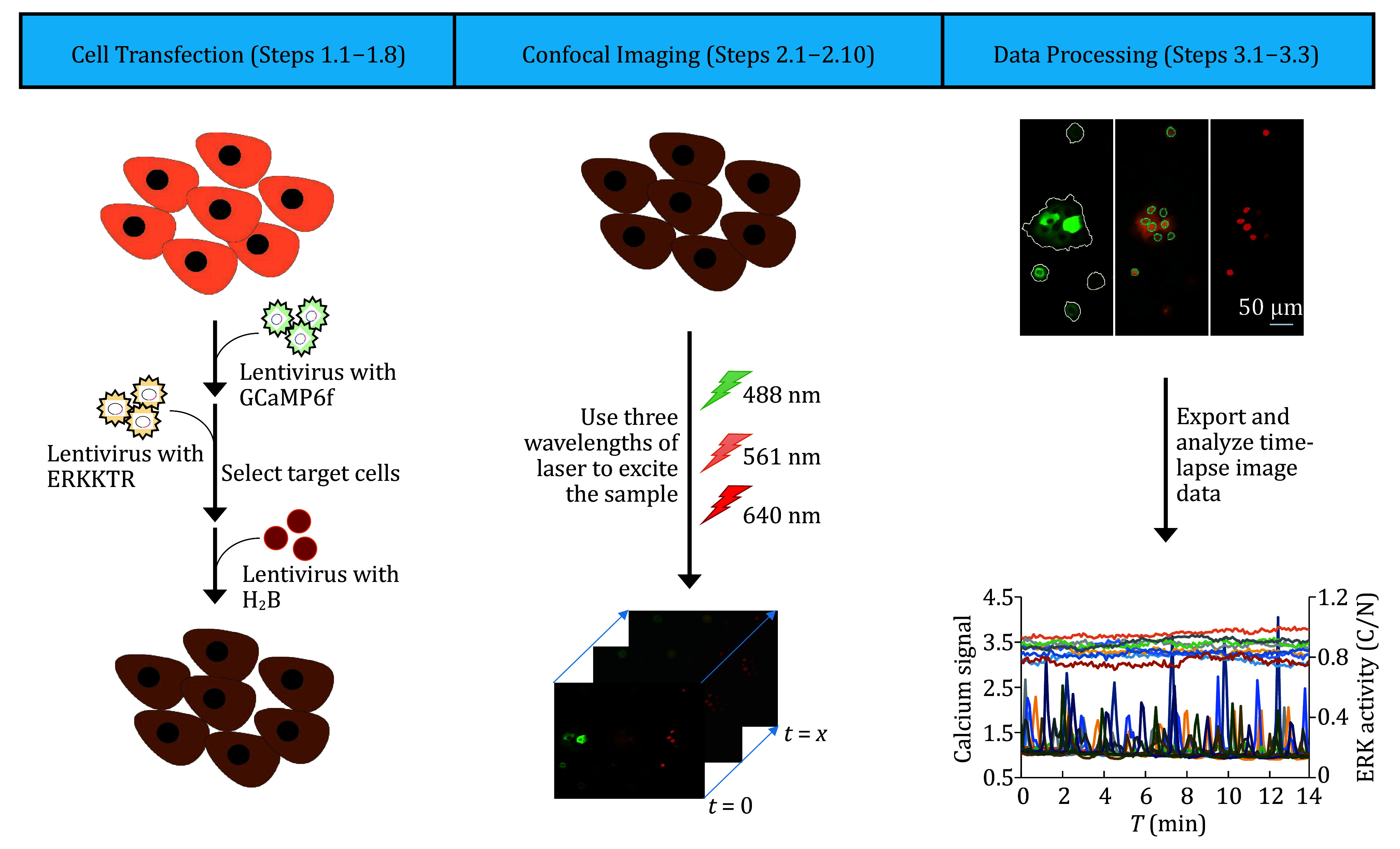
General schematic showing how calcium signal and ERK activity are simultaneously detected in living cells. First, three lentiviruses containing corresponding probes were constructed to infect cells separately in order. Drug screening was performed after the second infection. Infected cells were imaged with a confocal microscopy in three wavelengths of lasers. Finally, the calcium signal and ERK activity were measured and integrated into a line graph

The entire plan includes material preparation and experimental procedures. The detailed experimental procedures were divided into three consecutive stages: introduction of three fluorescent probes into cells, living-cell confocal microscopy imaging, and data processing of calcium signal and ERK activity (Fig. 1).

## MATERIALS, INSTRUMENTATION AND SOFTWARE

### Probe construction

The GCaMP6f fragment located at 1166-2518 bp was cloned from the plasmid pGP-CMV-GCaMP6f (Addgene, cat. no. 40755) and transferred into the constructed lentivirus vector Lenti-Last-Vector (blasticidin resistance in eukaryotic cells) to package lentivirus plasmid pLKO.1-GCaMP6f-blast. Plasmids pLentiPGK-Blast-ERKKTRmRuby2 (Addgene, cat. no. 90231, blasticidin resistance in eukaryotic cells) and pLentiPGK-DEST-H2B-iRFP670 (Addgene, cat. no. 90237) were directly packaged as lentiviruses. The above three types of lentiviruses were all concentrated into 1 × 10^8^ transducing units (TU) /mL titer, stored at –80 °C for future use.

### Cells, reagents and consumables

• NCI-H1650 cells (ATCC, cat. no. CRL-5883)

• Fetal bovine serum, FBS (Biochannel, cat. no. BC-SE-FBS01)

• Roswell Park Memorial Institute, RPMI-1640 (Gibco™, cat. no. 11875-093)

• Phenol free minimum essential medium, MEM (Gibco™, cat. no. 51200-038)

• Sodium pyruvate (Gibco™, cat. no. 11360-070)

• Penicillin-streptomycin (Gibco™, cat. no. 15140-122)

• Polybrane (5 mg/mL stock solution, GUANGZHOU IGE BIOTECHNOLOGY LTD, cat. no. 220121Y01)

• Blasticidin S HCL (Selleckchem, cat. no. S7419)

• Fibronectin human plasma (SIGMA, cat. no. F2006-5MG)

• Phosphate buffered saline, PBS (VivaCell, cat. no. C3580-0500)

• 6-well plate (Corning, cat. no. 3516)

• 35-mm glass dish (NEST, cat. no. 801001)

• 35-mm glass-like dish (Cellvis, cat. no. D35-14-1.5P)

• 12-well glass bottom plate (Cellvis, cat. no. P12-1.5H-N)

• 12-well glass-like bottom plate (Cellvis, cat. no. P12-1.5P)

### Reagent setup

• Complete culture medium: 10% FBS (*v*/*v*), 1 mmol/L sodium pyruvate, 100 U/mL penicillin-streptomycin, RPMI-1640

• Phenol-free medium for imaging: 1%FBS (*v*/*v*), MEM (Phenol-free)

### Equipment

• CO_2_ incubator (Thermo scientific, cat. no. 4111)

• Cell Counter (Olympus, cat. no. R1)

• Laser confocal microscope (Nikon, cat. no. A1R HD25)

• Fluorescence microscope (Nikon, cat. no. Ti2)

• Small gas supplied incubator (Tokai Hit, cat. no. STXG-WSKMX-SET)

• Centrifuge (Dynamica, VELOCITY14R)

• Clean bench (AIRTECH, cat. no. SW-CJ-1FD)

### Software

• NIS-Elements AR Ver. 5.01

• GraphPad Prism Ver. 8

## PRPCEDURE

### Step 1: Introduction of GCaMP6f, ERKKTR and H_2_B probes into cells [TIMING 4–5 weeks]

Step 1.1: H1650 cells were inoculated into a six-well plate (1.5 × 10^5^ cells per well) with a final density of about 30% confluence. Add 2 mL of complete culture medium to each well and incubate the plate overnight in a 37 °C incubator supplied with 5% CO_2_.

Step 1.2: Wash the cells twice with preheated serum-free culture medium and then add complete culture medium with 8 μg/mL polybrane. Subsequently, add 15 μL (10 TU virus/cell) pLKO.1-GCaMP6f-blast lentivirus into the cell culture medium of each well, gently shake the plate, and place it overnight in a 37 °C incubator supplied with 5% CO_2_.

**[CRITICAL STEP]** It is necessary to try different virus titers within the range of 5–50 TU viruses/cells to find the optimal transfection efficiency and ensure that the cells are in a healthy state.

Step 1.3: Remove the original culture medium, wash the cells twice with serum-free culture medium, and add 2 mL of complete culture medium to each well. Let the cells recover in a 37 °C incubator for about a week, during which the culture medium is changed and the cells are subcultured every two days.

**[? TROUBLESHOOTING]**

Step 1.4: After recovery cultivation, repeat above Steps 1.1–1.3 to infect the cells with pLentiPGK-Blast-ERKKTRmRuby2 lentivirus.

**[? TROUBLESHOOTING]**

Step 1.5: During the subsequent subculture process, add 10 μg/mL blasticidin into the complete culture medium for cellular drug screening. Change the fresh culture medium containing 10 μg/mL blasticidin daily, and subcultured cells every two days. After one week of cultivation (three cell passages), a complete culture medium without drugs was used to replace the current medium for further restoration of culture.

Step 1.6: Seed 1 × 10^5^ infected cells into a 35-mm glass bottom culture dish, and incubate the dish overnight in a 37 °C incubator supplied with 5% CO_2_. Cells were examined under a fluorescence microscope. The excitation and corresponding emission filters are set as follows: Ex-480/30 nm (465–495 nm) and Em-535/46 nm (512–558 nm) for GCaMP6f; Ex-545/25 nm (533–558 nm) and Em-605/70 nm (570–640 nm) for ERKKTR. The proportion of cells carrying both GCaMP6f and ERKKTR probes should exceed 65%.

**[CRITICAL STEP]** It should be confirmed that there is a major proportion of infected cells (>65%) carrying corresponding two probes. If the proportion is low, refer to the troubleshooting section below.

**[? TROUBLESHOOTING]**

Step 1.7: After recovery cultivation, repeat above Steps 1.1–1.3 to infect the cells with pLentiPGK-DEST-H_2_B-iRFP670 lentivirus.

**[? TROUBLESHOOTING]**

Step 1.8: Seed 1 × 10^5^ the infected cells into a 35-mm glass bottom culture dish for the second round of microscopic examination. Filter settings for excitation and emission of H_2_B probe: Ex-605/50 nm (580–630 nm) and Em-670/50 nm (645–695 nm). The proportion of cells carrying GCaMP6f, ERKKTR and H_2_B probes should exceed 50%, indicating that the first stage of the experiment is successful. Then further experimental research can be conducted.

**[? TROUBLESHOOTING]**

### Step 2: Living-cell confocal microscopy imaging [TIMING 24–36 h]

Step 2.1: Add 500 μL of PBS solution with 10 μg/mL fibronectin of each well to a 12-well plate with glass bottom. Then place the plate in a 37 °C incubator for about 5 h, with the aim of better adhesion of cells on the bottom of the plate.

Step 2.2: Discard the solution from each well and wash twice with PBS. Then seed each well with 5 × 10^4^ cells (with a density of about 20%), add 1 mL complete culture medium, and slowly shake well to mix the medium and cells. Then incubate the plate overnight in a 37 °C incubator with supplied with 5% CO_2._

Step 2.3: Discard the medium from each well, wash the cells twice with 37 °C serum-free medium, and add 1 mL of phenol-free medium with 1% FBS to each well. Then place the plate in a 37 °C incubator for at least 30 min.

**[CRITICAL STEP]** Cell imaging experiments should use a phenol-free culture medium to reduce the interference of the fluorescence background.

**[NOTE]** During the microscopy imaging experiment, all reagents in contact with cells should be preheated to 37 °C in advance.

**[? TROUBLESHOOTING]**

Step 2.4: Confocal imaging is achieved using a Nikon A1R HD25 confocal microscope with a 40×/1.25 N.A. water immersion objective. Start the machine 30 min before the experiment. Pre-start a confocal associated small incubator (Tokai Hit) supplied with 5% CO_2_ and set it to a constant temperature of 37 °C, 100% humidity.

Step 2.5: Quickly transfer the pretreated plate with the cells from the normal incubator to the confocal associated small gas-supplied incubator, put the lid on, and let the plate stand for about 30 min. Wait until the cell state stabilizes before imaging.

Step 2.6: Use the following excitation lasers and corresponding emission filter when shooting: GCaMP6f probe (Excitation: 488 nm; Emission: 500–550 nm), ERKKTR probe (Excitation: 561 nm; Emission: 570–620 nm) and H_2_B probe (Excitation: 640 nm; Emission: 663–738 nm).

Step 2.7: Turn on the "Perfect Focus System" to prevent the focus drift, and find the appropriate focal plane by adjusting the Z direction to ensure clear cell contours. Adjust the X/Y direction and select healthy cells with all three fluorescent probes expressed for imaging.

**[CRITICAL STEP]** Hardware or software that is functionally similar to PFS must be used to ensure the stability of the focal plane in long-term (over 2 h) live cell photography.

Step 2.8: The confocal pinhole is set to 96 μm, which has an optical cross-section of about 2 μm at a wavelength of 488 nm. According to experimental conditions such as cell conditions and image signal-to-noise ratio, appropriate adjustments are made to parameters such as laser input, detector gain and amplification factor. Follow-up experiments should keep the same parameters for reference.

**[CRITICAL STEP]** Overexposure should be avoided by adjusting parameters such as laser input or detector gain in an appropriate value, otherwise it may lead to inaccurate subsequent measurements.

**[? TROUBLESHOOTING]**

Step 2.9: Use bidirectional scanning mode, set 0.7 s/frame (512 × 512 pixels) with 6 s intervals to capture fluorescence signals that rapidly and dynamically change over time. The continuous imaging time is determined according to the experimental needs, usually ranging from 30 min to 8 h.

**[? TROUBLESHOOTING]**

Step 2.10: When dosing the drug, pause the imaging process, open the lid, and use the pipette to add preheated drug solution slowly and evenly. Then cells can be left to stand for 10–30 min according to the experimental arrangement. In addition to directly opening the lid to add drugs, a designed perfusion system can also be used for drug dosing during continuous photography.

**[? TROUBLESHOOTING]**

### Step 3: Data processing of calcium signal and ERK activity [TIMING 3–5 h]

#### Step 3.1: Denoise and subtract background

Step 3.1.1: Open the Image with NIS-Elements AR software to process the digital images, Convert and select the “Change Color Depth” option. Convert the file to 12-bit data.

**Figure 2:**
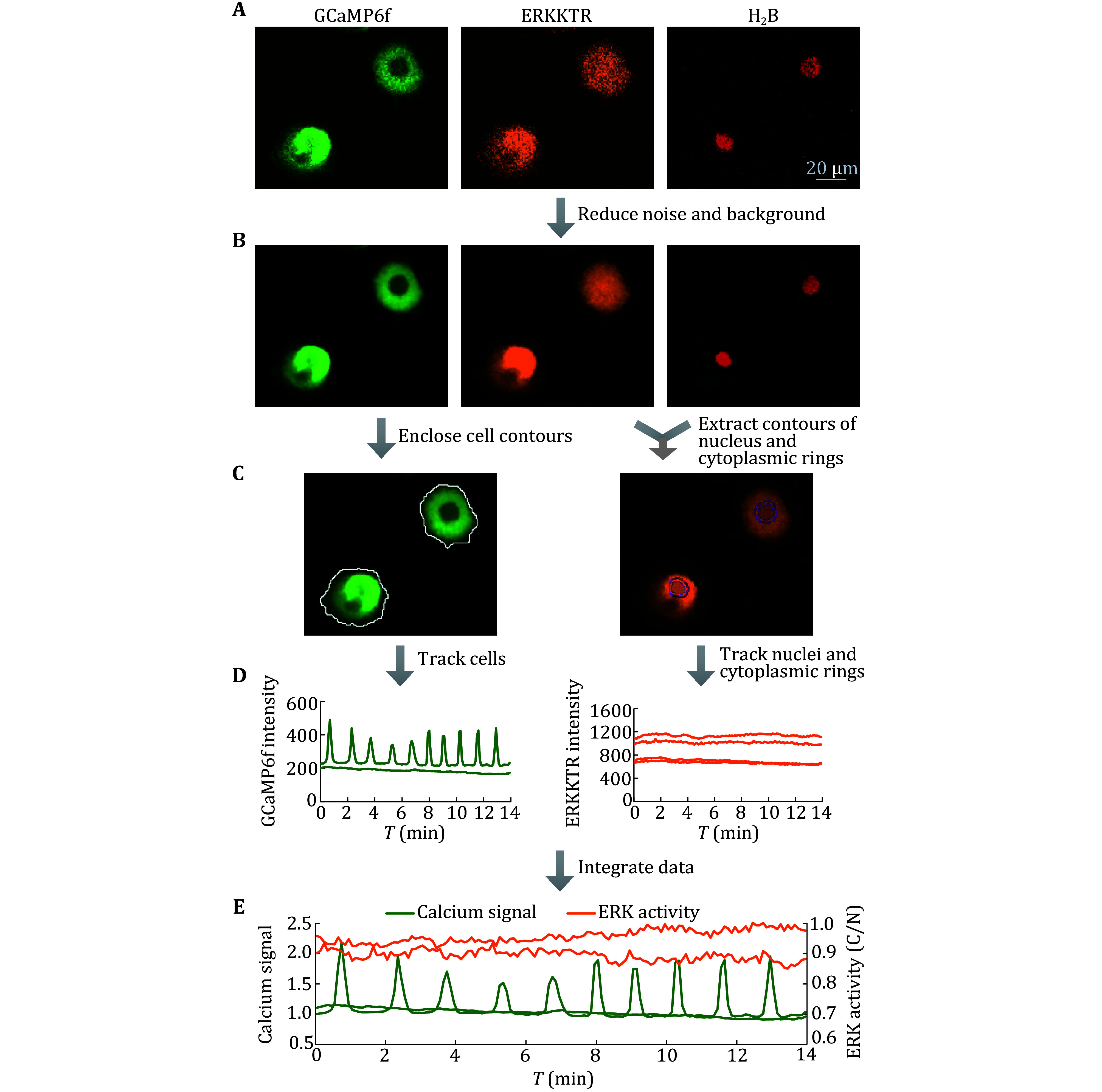
Image processing and data analysis of GCaMP6f, ERKKTR and H_2_B channels. **A** Initial fluorescent images of GCaMP6f (left), ERKKTR (middle), and H_2_B (right) channels. **B** Processed fluorescent images of three channels through noise reduction and background removal. **C** Left, Separately extracted GCaMP6f channel image with clear cell contours after threshold processing. Right, ERKKTR channel with the counters of the cytoplasmic ring and nuclear region. The nuclear region was determined by the H_2_B channel. **D** Raw data of calcium signal (left) and ERK activity (right). **E** Integrated line graph with the normalized calcium signal and the calculated ERK activity

Step 3.1.2: Click the “Advanced Denoising” option in the “Image” menu, select GCaMP6f, ERKKTR and H_2_B fluorescence channels for noise reduction with the denoising power of 30%, and apply to all frames (Figs. 2A and 2B).

Step 3.1.3: Use “Maximum Intensity Projection” to overlay the image, select an area without cells or impurities in all channels, and circle the area as the background ROI (regions of interest) with the “Draw Circular ROI” in the “ROI” menu. Select the “Background” option in the “Image” menu and use the background ROI to subtract the fluorescence background (Figs. 2A and 2B).

#### Step 3.2: Calcium signal processing

Step 3.2.1: Extract GCaMP6f channel images separately, select the “Define Threshold” option in the “Binary” menu, and adjust the threshold in the appropriate range to make sure the proper cell contours (Fig. 2C, left). Meanwhile, check “Size” and set a proper value to remove non-living cell impurities, select “Smooth” at 1×, open the “Fill holes” option, and apply it to all frames.

Step 3.2.2: Select the “Tracking” option in the “Analysis control” menu, set relevant parameters to adapt to the currently analyzed file, such as checking random motion and constant speed, setting the maximum gap size to 3, and setting the standard deviation multiplication factor to 3.

Step 3.2.3: Click on the “Tracking” button to obtain multiple parameters that change over time within the cell contours. In the graph, select the “MeanIntensity” value in the “left X-axis” option and export the data to Excel. The data are the original fluorescence values of calcium signals (Fig. 2D, left).

Step 3.2.4: To calculate the *F*/*F*_0_ value of the calcium signal, the baseline fluorescence intensity (*F*_0_) is determined by the median of the “MeanIntensity” during the period of no calcium signal in Execl. Divide the “average intensity” of the calcium signal (*F*) at each time point by the baseline fluorescence intensity (*F*_0_) to obtain the normalized data of the calcium signal (*F*/*F*_0_) over time (Fig. 2E).

#### Step 3.3: ERKKTR data processing

Step 3.3.1: Extract H_2_B and ERKKTR channels. In the H_2_B channel, select the “Define Threshold” option in the “Binary” menu and set an appropriate threshold range to determine the nucleus contour represented by H_2_B, also check “Size” and set a suitable range value to ensure the exclusion of non-living cell impurities.

Step 3.3.2: Right click on the image and select the “Copy Binary Layers” option. Copy the binary layer of the H_2_B channel three times onto the ERKKTR channel, named "Original", "Intra-nuclear" and "Cytoplasm", respectively.

Step 3.3.3: In the “Binary” menu, select the “Erode” option to shrink 1–2 pixels of the "Intra-nuclear" layer to ensure that it is completely located within the nucleus and does not contain cytoplasmic regions. Select the “Dilate” option to expand the "Cytoplasm" layer 1–2 pixels to ensure that the cytoplasmic area around the nucleus is generated.

Step 3.3.4: Use the “Binary Operations” function in the “Binary” menu to subtract the "Original" layer from the "Cytoplasm" layer, generate a "Subtraction" layer, which is the cytoplasmic ring, and rename the "Cytoplasmic ring" layer.

Step 3.3.5: Track the "Cytoplasmic ring" layer and "Intra-nuclear" layer (Fig. 2C, right), select the “MeanIntensity” value in the “left X-axis” option in the graph, and export the data to Excel (Fig. 2D, right).

Step 3.3.6: To calculate the C/N parameter of ERKKTR, the mean intensity of the "Cytoplasmic ring" layer (C) is divided by the mean intensity of the "Intra-nuclear" layer (N) of the corresponding frame in Excel to obtain the ratio value, which is the C/N parameter reflecting the ERK activity (Fig. 2E).

**[CRITICAL STEP]** Since the C/N ratio is calculated by dividing the mean intensity of cytoplasm by the mean intensity of the nucleus during ERKKTR data processing, it inevitably leads to difficulties in quantitatively measuring the value under certain conditions, such as very low probe expression, significant changes in cell state or morphology (cell division or detaching), or cell movement out of the imaging frame. Therefore, suitable cells should be selected according to the experimental needs, and the apoptotic, dividing, floating and untraceable cells should be deleted.

**[TIMING]**

Step 1, cell infection: 4–5 weeks

Step 1.1, cell seeding and incubation: ~12 h

Step 1.2, cell infection of GCaMP6f lentivirus: ~12 h

Step 1.3, changing culture medium: ~30 min; Cell restore culture: ~1 week

Step 1.4, cell infection of ERKKTR lentivirus and restore culture: ~1 week

Step 1.5, cellular drug screening with blasticidin: ~1 week

Step 1.6, cell seeding, incubation and microscopic detection of infection efficiency: ~18 h

Step 1.7, cell infection of H_2_B lentiviruses and restore culture: ~1 week

Step 1.8, second round microscopic detection of infection efficiency: ~18 h

Step 2, living-cell confocal microscopy imaging: 24–36 h

Step 2.1, fibronectin incubation on imaging culture plate: ~5 h

Step 2.2, cell implantation and incubation: ~12 h

Step 2.3, replacing the culture medium with phenol-free medium: ~30 min

Step 2.4, instrument preparation: ~30 min

Step 2.5, cell incubation to the stable state: ~30 min

Steps 2.6–2.8, parameters setting for laser confocal microscope: ~30 min

Steps 2.9–2.10, living cell imaging: 0.5–8 h

Step 3, tracking and analysis of imaging data: 3–5 h

Step 3.1, denoise and subtract background: ~30 min

Step 3.2, calcium signal processing: 1–2 h.

Step 3.3, ERKKTR data processing: 1–2 h.

**[? TROUBLESHOOTING]**

Troubleshooting advice can be found in Table 1.

**Table 1:** Troubleshooting table

Step	Problem	Possible reason	Solution
1.3, 1.4, 1.7	Unhealthy cells after infection, floating, or apoptotic	(1) Insufficient recovery cultivation time; (2) Excessive amount of infected virus	(1) Increase the interval culture time; (2) Appropriately reduce the amount of virus
1.6, 1.8	Low infection efficiency	(1) Insufficient virus titer; (2) Low efficiency of genome integration after infection	(1) Properly increase the virus titer; (2) Selecting monoclonal clones; (3) Adding different drug screening genes to virus vectors; (4) Replace infected virus H_2_B-iRFP670 with loading SIR-DNA before imaging
2.3	Few adherent cells after passage before live cell imaging	Inappropriate coating material or culture dish material	(1) Increase the incubation time and concentration of fibronectin; (2) Replace the fibronectin with collagen or laminin; (3) Change the culture dish to glass like material (Cellvis, cat. no. P12-1.5P).
2.8, 2.9	The fluorescence signals of different channels undergo non-specific co-localization or overlap	Fluorescent channels crosstalk	(1) Application sequence scanning mode; (2) Reduce the laser power of the interfering channel, increase its detector gain; (3) Increasing the laser power of the interfered channel and reducing its detector gain
2.9	The overall fluorescence intensity shows a significant decrease over time	Fluorescence photobleaching	Reduce laser power, increase detector gain, or increase sampling interval
2.10	Focus drift after drug dosing.	Vibration caused by opening and closing the lid	(1) Using pre prepared hoses and syringes for drug dosing without opening the lid; (2) Dosing through additional perfusion system

## ANTICIPATED RESULTS

**Figure 3:**
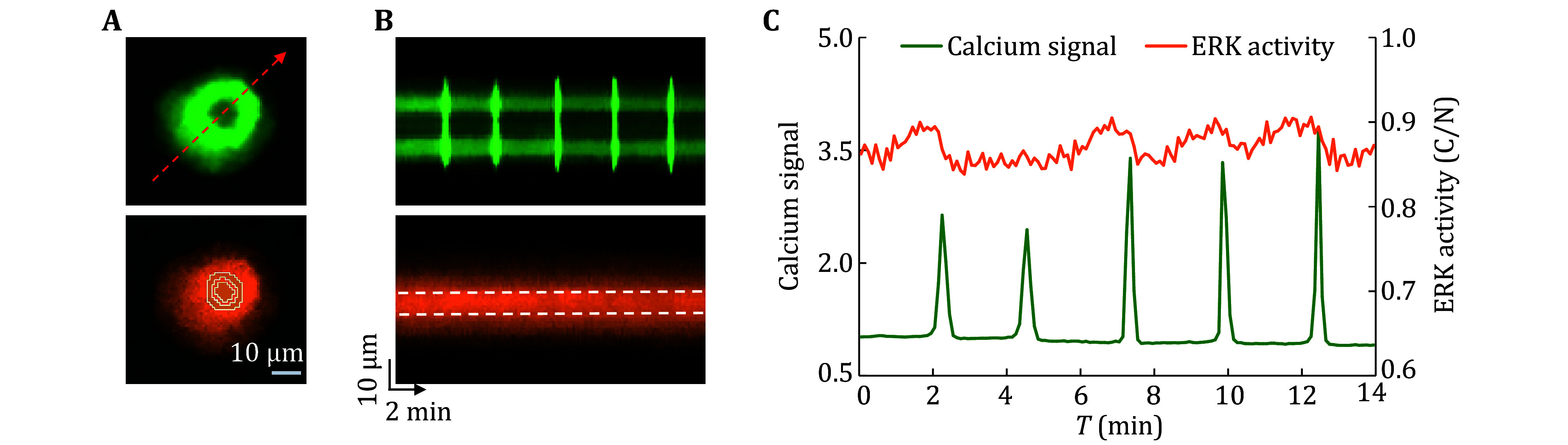
Calcium signal and ERK activity of a quiescent single cell. **A** Top, GCaMP6f channel of a quiescent epithelial H1650 cell. The line passing through the nucleus denotes the Kymograph line position. Bottom, ERKKTR channel of the quiescent single cell with the counters of the cytoplasmic ring and nuclear region. **B** Kymographs images created at the location of dash lines in Panel A, which display the calcium signal and ERKKTR signal of the cell over time. The white dashed line represents the nucleus region in the ERKKTR kymograph. **C** Integrated line graph with the cellular calcium signal and ERK activity

**Figure 4:**
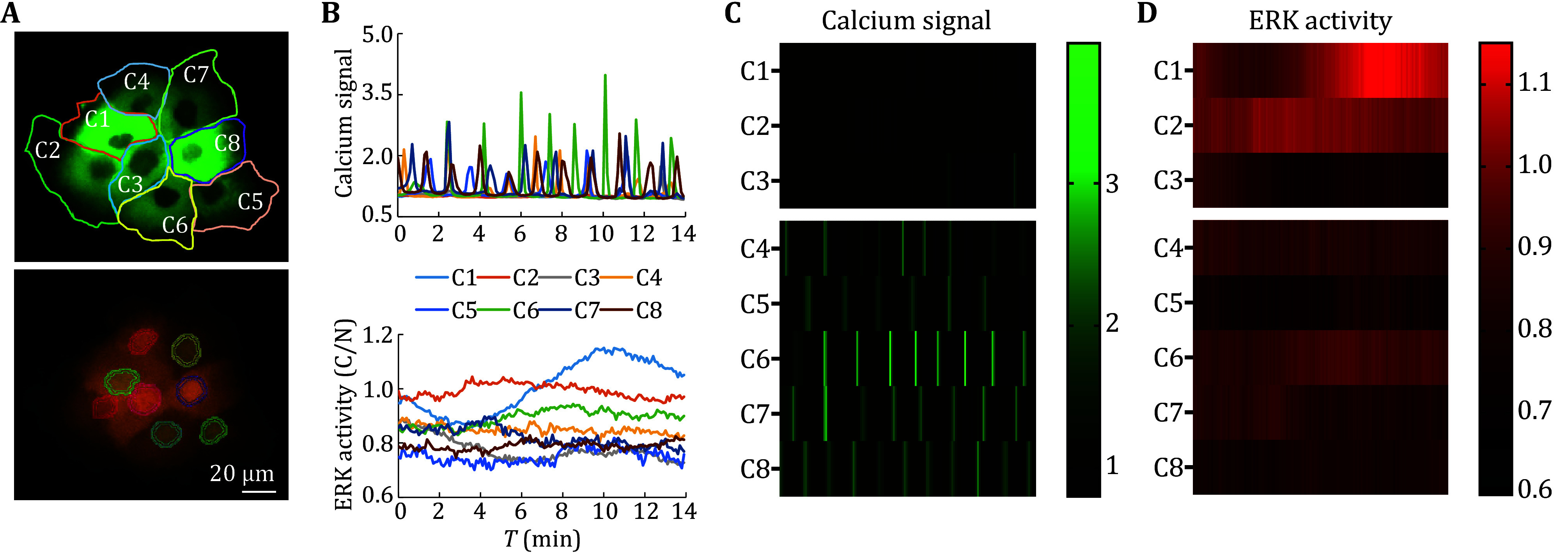
Calcium signal and ERK activity of the clustered cells. **A** Top, GCaMP6f channel of the clustered epithelial H1650 cells with drawn edges using polygonal ROI and marked with numbers (C1–C8). Bottom, ERKKTR channel is marked with cytoplasmic rings and nuclear regions. **B** Line polts of calcium signal (top) and ERK activity (bottom) in individual cells within the cluster. **C**,**D** Heatmap of the calcium signal (**C**) and corresponding ERK activity (**D**) in individual cells within the cluster. The heatmap data were sorted by the intensity and frequency of the cellular calcium signal

**Figure 5:**
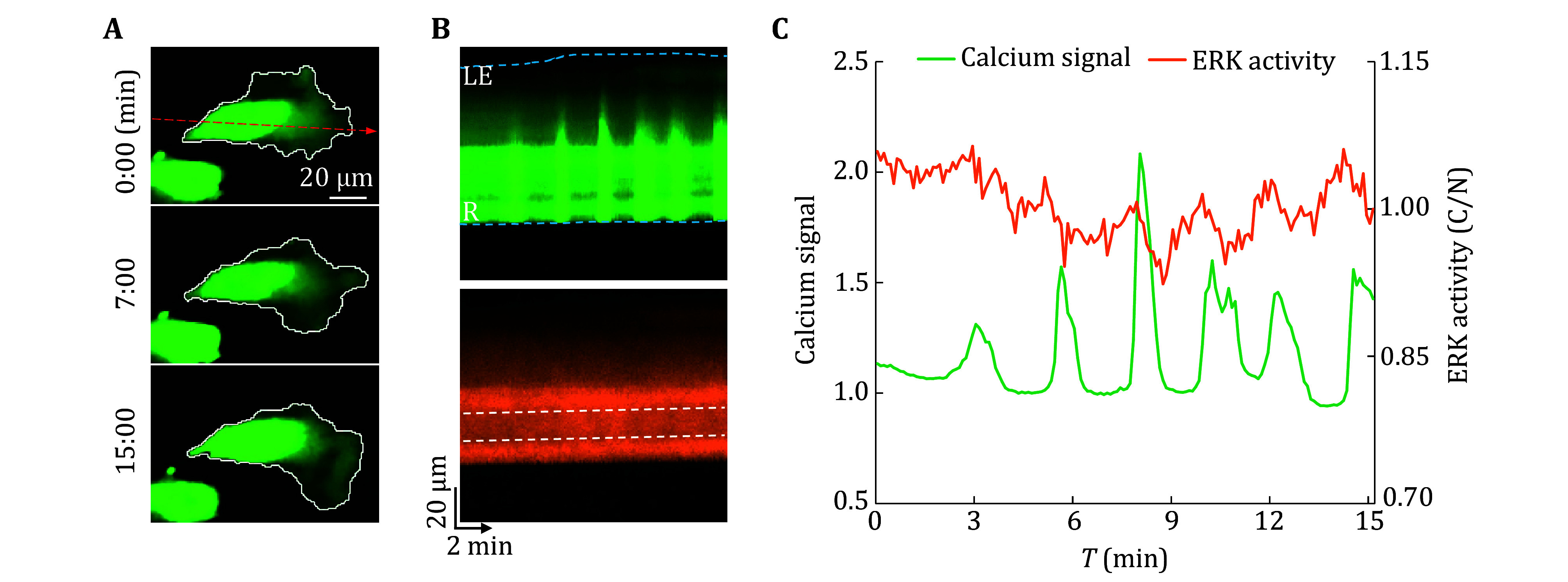
Calcium signal and ERK activity in migrating cells. **A** The GCaMP6f channel of a migrating mesenchymal H1650 cell at different time points. White lines denote cell contours. The red line passing through the nucleus denotes the Kymograph line position. **B** Kymographs images of GCaMP6f channel (top) and ERKKTR channel (bottom) from the migrating cell at the location of dash lines in Panel A. The blue dashed line in the GCaMP6f kymograph represents the continuously moving cell boundaries. The white dashed line represents the nucleus region in the ERKKTR kymograph. LE denotes the leading edge and R denotes the rear. **C** A line chart showing both dynamic calcium signal and ERK activity of the migrating cell

Due to synchronous detection based on stable expression of fluorescent proteins in this scheme, after optimizing relevant experimental conditions and parameters based on cell density, type and state, the protocol can be applied to dynamic imaging of living cells at different spatiotemporal scales. Based on the time-lapse series imaging results, it was possible to simultaneously track calcium signals and ERK activity in a single quiescent cell at high temporal resolution (second level) (Figs. 3A and 3B). Further analysis of the spatiotemporal characteristics of both could observe a small ERK fluctuation signal that lasted for about two minutes before most calcium transients (Fig. 3C). In a cell cluster, individual cells could be distinguished based on the nucleus (Fig. 4A). By combining the background fluorescence intensity of other channels, region segmentation and computational tracking on each cell could be performed on each cell to achieve simultaneous tracking and detection of calcium signals and ERK activity of individual cell in the clusters (Figs. 4A and 4B), and to determine communication and interaction of signal networks between cells. Taking the cluster of H1650 epithelial tumor cells as an example, two types of cells with different dynamic characteristics could be found: one group of cells (C1–C3) had little or no calcium transients (Fig. 4C), while ERK activity exhibited significant oscillations over time (for example, ERK activity in C1 oscillated from 0.86 to 1.15) (Fig. 4D); Another group of cells (C4–C8) had high-frequency calcium transients, but their ERK activity was relatively stable. In addition, due to the separate detection and tracking of each image in the series by the processing software, it is also suitable for detecting cells in a migratory state (Fig. 5), dynamically detecting the spatial distribution of calcium signals and changes in ERK activity, and revealing the characteristics and interactions of the two signals under different movement modes such as collective migration or single cell movement. Taking the mesenchymal H1650 cell as an example (Fig. 5A), local calcium transients raised from the rear could not propagate to the leading edge (Fig. 5B, top), while small ERK activity pulses appeared after those local calcium transients (Figs. 5B and 5C).

Moreover, combining this protocol with the application of pharmacological stimulants or inhibitors can quickly provide accurate and easily reproducible experimental results to determine the characteristics, causality, and interaction of two signals in cells under different physiological or pathological conditions. If the calcium probe is modified and specific organelle localization sequences are introduced, combined with super-resolution imaging technology, precise localization and detection of organelle scale calcium signals can be achieved, and the interaction between calcium signals and ERK signals at the subcellular level can be explored. Based on the wide adaptability of KTR probes, the detection part of ERK activity can be replaced by protein sequences that are sensitive to other kinase molecules (such as JNK, p38, or PKA, *etc*.), which enables synchronous and dynamic detection of calcium signals and other signaling pathways in living cells.

## Conflict of interest

Liting Zhang, Yan Mo, Shimin Mo, Ming Xia and Chaoliang Wei declare that they have no conflict of interest.
